# DESIGN NEEDS AND PERCEPTIONS OF OLDER ADULTS REGARDING SHARED-USE AUTOMATED VEHICLES

**DOI:** 10.1093/geroni/igad104.2864

**Published:** 2023-12-21

**Authors:** Justin Lam, Tarryn O’Rourke, Caitlin Bowman, Clive D’Souza

**Affiliations:** Carnegie Mellon University, Pittsburgh, Pennsylvania, United States; University of Pittsburgh, Pittsburgh, Pennsylvania, United States; University of Pittsburgh, Pittsburgh, Pennsylvania, United States; University of Pittsburgh, Pittsburgh, Pennsylvania, United States

## Abstract

Many communities struggle to provide safe, accessible, and reliable transportation services for older adults due to high demand, rising costs, driver shortages, and other evolving challenges. Innovative transportation solutions are needed to support the current and future populations of older adults. Low-speed, shared-use, driverless shuttles present an exciting development in automated vehicle (AV) technology with potential to meet mobility needs of older adults in their community. Understanding older adults’ perceptions about and willingness to consider using these emerging modes of transportation is vital to realizing the full potential of these technologies. This presentation summarizes an in-person study conducted with 12 older (average: 66 +/- 4 years of age, range: 60 to 80 years) and 10 younger (average: 44 +/- 11 years) adults that evaluated a stationary, proof-of-concept shared-use AV retrofitted with accessibility features. We will present findings on perceptions regarding accessibility, safety, and willingness to use driverless AVs along with human factors design recommendations. While questionnaire-based studies have been the dominant approach to understanding older adults’ perceptions about shared-use AVs, in-person evaluations even with prototype AVs as described here, provide opportunities to identify goals, needs and preferences of older adults concerning usability and safety in early design stages, and through hands-on exploration help older adults develop good mental models, i.e., understand AV capabilities and limitations, towards building trust and acceptance for these emerging modes of transportation. Research and policy implications will be discussed towards enabling emerging driverless shared-use AV technologies that support safe and independent community mobility for older adults.

